# Enhancing Medical Education Through Statistics: Bridging Quantitative Literacy and Sports Supplementation Research for Improved Clinical Practice

**DOI:** 10.3390/nu17152463

**Published:** 2025-07-28

**Authors:** Alexander A. Huang, Samuel Y. Huang

**Affiliations:** 1 Department of Computer Science, Cornell University, Ithaca, NY 14850, USA; 2 Feinberg School of Medicine, Northwestern University, Chicago, IL 60611, USA; 3 School of Medicine, Virginia Commonwealth University, Richmond, VA 23298, USA

**Keywords:** statistics, education, machine learning, medical education

## Abstract

In modern medical education, a robust understanding of statistics is essential for fostering critical thinking, informed clinical decision-making, and effective communication. This paper explores the synergistic value of early and continued statistical education for medical students and residents, particularly in relation to the expanding field of sports supplementation and its impact on athletic performance. Early exposure to statistical principles enhances students’ ability to interpret clinical research, avoid cognitive biases, and engage in evidence-based practice. Continued statistical learning throughout residency further refines these competencies, enabling more sophisticated analysis and application of emerging data. The paper also addresses key challenges in integrating statistics into medical curricula—such as limited curricular space, student disengagement, and resource constraints—and proposes solutions including interactive learning, case-based teaching, and the use of public datasets. A unique emphasis is placed on connecting statistical literacy to the interpretation of research in sports science, particularly regarding the efficacy, safety, and ethical considerations of sports supplements. By linking statistical education to a dynamic and relatable domain like sports performance, educators can not only enrich learning outcomes but also foster lasting interest and competence in quantitative reasoning. This integrated approach holds promise for producing more analytically proficient and clinically capable physicians.

## 1. Introduction

In the evolving landscape of modern medicine, the ability to critically assess research and apply evidence-based knowledge is vital for effective clinical practice [1,2,3]. At the heart of this competency lies statistical literacy—a foundational skill often underemphasized in traditional medical education [4]. Medical students frequently perceive statistics as abstract and disconnected from clinical relevance, leading to disengagement and the underutilization of a critical skillset [5,6]. Without a solid grounding in statistics, future physicians may struggle to interpret medical literature, assess risk accurately, or apply clinical guidelines with confidence.

One promising strategy to address this disconnect is to contextualize statistical instruction in domains that naturally capture student interest and demonstrate practical utility. Sports supplementation—an increasingly prominent topic in both public health and clinical conversations—offers an ideal entry point for this integration [7,8]. The growing popularity of performance-enhancing supplements among athletes and the general population creates a clear need for physicians to evaluate claims of efficacy, understand potential harms, and consider ethical concerns grounded in the current evidence base [9,10,11,12]. Performing this requires not only domain-specific knowledge but also the ability to interpret quantitative data, appraise the quality of study designs, and communicate findings clearly to patients.

By anchoring statistical concepts in the real-world context of sports supplementation, educators can enhance student engagement while improving applied learning outcomes. This integration enables students to practice interpreting clinical trials, performing critical appraisal, and contextualizing statistical results within patient-centered care. Moreover, teaching statistics through such tangible, relevant content may help overcome the perception of statistics as isolated from the clinical realities students will face during training and practice [13,14].

This paper explores the intersection between statistical education and sports supplementation research to propose a more dynamic and engaging model of statistical instruction in medical curricula. We discuss the benefits of early and longitudinal exposure to statistical reasoning, identify persistent barriers to effective teaching, and outline evidence-based pedagogical strategies—including the use of public datasets, interactive case studies, and clinically relevant modules—that align with the cognitive needs and motivations of medical students [15,16]. Through this integrated approach, we aim to support the cultivation of analytically competent physicians equipped to deliver high-quality, evidence-informed care across medical specialties [17].

## 2. Methods

This paper utilizes a narrative review and conceptual analysis to explore how statistical education can be effectively integrated into medical training, particularly through its application to sports supplementation research. A literature review was conducted using academic databases such as PubMed, MEDLINE, ERIC, and Google Scholar. Search terms included medical statistics education, statistical literacy in medicine, sports supplements, sports nutrition, evidence-based practice, and medical education curriculum. The review focused on peer-reviewed publications, educational models, and guidelines from the last 15 years to ensure the relevance and currency of the findings.

A thematic synthesis was then performed to identify and organize key themes emerging from the literature. These included the recognized benefits of both early and continued statistical education for medical students and residents, the common challenges faced in integrating statistics into existing medical curricula, and effective strategies for promoting engagement—especially through practical, clinically relevant applications. Special attention was given to how real-world research on sports supplements can serve as a vehicle for reinforcing key statistical concepts such as confidence intervals, *p*-values, statistical inference, and hypothesis testing.

To support this analysis, a case integration approach was employed, incorporating selected examples from published research studies on widely used sports supplements such as creatine, caffeine, and beta-alanine. These case studies served to demonstrate how students could apply statistical tools to critically evaluate supplement efficacy and safety, thereby bridging theoretical knowledge with clinical relevance. By grounding statistical lessons in tangible, high-interest topics, this approach aimed to increase student motivation and comprehension.

A pedagogical framework review was also undertaken to evaluate current educational strategies that successfully incorporate statistics into medical curricula. This review examined teaching models such as problem-based learning (PBL), case-based learning (CBL), and the use of public datasets for interactive data analysis exercises. Emphasis was placed on methods that promote active learning and sustained student interest in statistical reasoning. The use of publicly available datasets was especially highlighted for its potential to provide realistic, hands-on learning experiences that mirror actual research and clinical evaluation processes.

Lastly, informal expert consultations were conducted with educators in biostatistics, medical education, and sports medicine to assess the relevance and feasibility of the proposed integration. While not part of a formal research protocol, these consultations offered valuable insights and validation of the conceptual connections and teaching strategies presented in the paper.

## 3. Results

The analysis of literature and pedagogical models yielded several key findings that support the integration of statistical education with sports supplementation research in medical training. First, the literature review confirmed that early exposure to statistics in medical school significantly improves students’ ability to interpret clinical research, make evidence-based decisions, and recognize methodological flaws such as bias and confounding. Continued statistical education during residency further strengthens these skills, enhancing diagnostic accuracy, treatment planning, and research competence. However, challenges such as time constraints, limited faculty expertise, and student disengagement persist across many institutions.

From the thematic synthesis, it was evident that one of the most effective ways to overcome these barriers is through contextual learning. Integrating statistical instruction with topics that are perceived as practical and interesting—like sports supplementation—can increase both comprehension and engagement. Students are more likely to appreciate the value of statistical thinking when it is directly tied to real-world clinical and research questions. For instance, the analysis of studies on creatine, caffeine, and beta-alanine provided concrete examples of how to evaluate effect sizes, interpret confidence intervals, and assess study design quality.

The case integration approach demonstrated how research on sports supplements could serve as a powerful educational tool. For example, randomized controlled trials on creatine use in athletes allowed for in-depth exploration of hypothesis testing, *p*-value interpretation, and critical appraisal of sample size and power calculations. Similarly, observational data on supplement safety in general populations opened opportunities for teaching concepts such as correlation vs. causation and confounding variables. These cases showed clear potential to transform statistical concepts from abstract theory into clinically relevant skills.

The pedagogical framework review identified several instructional strategies that enhance student engagement with statistics. Problem-based and case-based learning approaches were especially effective in fostering active participation and the application of concepts. The use of public datasets, such as those from the CDC or NIH, provided accessible, real-world data for hands-on learning. Integrating sports nutrition and supplementation datasets into exercises enabled students to practice data analysis, hypothesis testing, and the interpretation of results in a context that was both relevant and motivating.

Finally, expert consultations supported the practical value of linking statistical instruction to sports supplementation. Educators noted that students often respond more positively when they can immediately see the clinical or lifestyle relevance of what they are learning. They also emphasized the growing importance of training future physicians to critically assess claims related to nutrition, fitness, and supplementation—areas increasingly intersecting with mainstream clinical care.

Together, these findings suggest that integrating statistical education with sports supplementation not only improves statistical literacy but also deepens clinical understanding and engagement, equipping future physicians with the tools to make more informed, data-driven decisions.

## 4. Discussion

The results of this review highlight the substantial potential of integrating statistical education with real-world applications—particularly through sports supplementation research—as a strategy to improve both medical training and clinical practice [16,18,19]. By illustrating how core statistical concepts such as hypothesis testing, regression modeling, and meta-analysis apply to evaluating supplement efficacy and safety, we present a compelling argument for embedding statistical instruction early and consistently throughout medical education [20,21]. This applied approach strengthens students’ ability to critically appraise research and promotes a deeper, more functional understanding of how statistical reasoning underpins sound clinical decision-making [16,17,22].

A central advantage of this integration lies in its ability to increase learner engagement by contextualizing abstract statistical concepts within familiar and relevant domains [20]. Sports supplementation represents a topic of growing public interest and clinical significance. When students evaluate the efficacy of widely used substances such as creatine or caffeine they are introduced to important statistical principles in a way that feels authentic and clinically useful. This connection helps overcome the common perception of statistics as a detached or inaccessible discipline [8,23,24]. Real-world data and case-based learning offer tangible entry points for students to see the immediate clinical relevance of statistical analysis—enhancing both motivation and retention.

Beyond initial exposure, the longitudinal reinforcement of statistical literacy through residency training is essential for cultivating analytic fluency [25,26,27,28]. In an era marked by a rapid growth in clinical trials and an expanding body of literature—including studies focused on emerging health behaviors like supplement use—physicians must be equipped with the tools to interpret both experimental and observational research [29]. Early and continuous training fosters habits of critical inquiry, empowering learners to assess bias, judge study validity, and evaluate the applicability of findings in varied clinical contexts [30].

Despite its promise, implementing integrated statistical education poses significant challenges. Chief among these is the limited curricular space in medical education, where content overload often precludes robust instruction in biostatistics [21]. Moreover, many students approach statistics with apprehension or disinterest, reinforced by the traditionally abstract nature of instruction [18,19,28,31]. However, pedagogical strategies that embed statistical learning into clinically aligned courses (e.g., pharmacology, biochemistry, or systems-based pathophysiology) offer a practical solution. Incorporating public datasets, interactive tools, and collaborative exercises can make statistical instruction more engaging and applicable. These active learning approaches support a deeper understanding and the long-term retention of key concepts [17,31,32].

This model of instruction has broader implications beyond the classroom. As the use of supplements grows among patients seeking athletic, metabolic, or general wellness benefits, physicians will increasingly be asked to interpret conflicting evidence and guide informed decision-making [3,13,33]. Clinicians who understand the statistical underpinnings of supplement studies—such as effect size, confidence intervals, and heterogeneity in meta-analyses—are better positioned to counsel patients on benefits, risks, and the strength of available evidence [18,20]. This reinforces the dual educational and clinical value of embedding statistical literacy in contexts that mirror real-world medical encounters.

Preliminary data from a small pilot workshop (n = 12) suggest that embedding supplement-focused case studies into biostatistics instruction can measurably enhance learners’ interpretive skills [24]. Post-session assessments demonstrated a 30% increase in accuracy when evaluating confidence intervals and heterogeneity in meta-analyses compared with the baseline [25]. To build on these findings, a structured implementation roadmap proposes quarterly, case-based modules targeting preclinical and clinical cohorts [26]. Predefined metrics—such as change in hypothesis-testing proficiency and regression-modeling accuracy—will guide ongoing evaluation [27]. A complementary pedagogical map visually links each core statistical concept (e.g., power analysis, multivariable regression, funnel-plot interpretation) to exemplar supplement studies [28]. This offers instructors and students an intuitive framework for applied learning and ensures consistency across instructional settings [29].

Despite the benefits of this applied approach, ethical complexities warrant careful consideration [30]. Using sports supplementation as a teaching exemplar risks unwittingly normalizing industry influence or medicalizing routine nutritional behaviors unless counterbalanced by explicit safeguards [31]. Mandatory disclosure of funding sources is essential to maintain transparency [32]. The integration of literature critical of industry sponsorship provides a balanced perspective [33]. Structured critical-appraisal exercises cultivate learner vigilance and scholarly integrity [34]. By foregrounding these practices, statistical instruction not only demystifies quantitative reasoning but also models the ethical scrutiny required for high-quality, evidence-informed clinical decision-making [35].

In summary, this review suggests that integrating statistical instruction with applied domains such as sports supplementation can meaningfully improve both educational outcomes and clinical preparedness [20]. Through early exposure, context-rich case studies, and sustained reinforcement across the medical education continuum, students gain not only technical knowledge but also the interpretive skills essential for high-quality, evidence-informed care [7,17,21,24,34]. Framing statistics through relevant and engaging content demystifies quantitative reasoning and equips future physicians to confidently navigate the increasingly data-driven landscape of modern medicine [15,28,32,35].

## 5. Conclusions

The findings suggest that while PAC utilization is associated with lower mortality rates in heart failure patients with ischemia, it is also linked to increased hospitalization costs and length of stay, indicating potential benefits even in specific populations where physiologically the data is less accurate.

## Data Availability

The original contributions presented in this study are included in the article. Further inquiries can be directed to the corresponding author.

## References

[1] Wu Y., Zhou L., Li G., Yi D., Wu X., Liu X., Zhang Y., Liu L., Yi D. (2015). Correction: Cognition of and Demand for Education and Teaching in Medical Statistics in China: A Systematic Review and Meta-Analysis. PLoS ONE.

[2] Webb R. (2018). Celebration, education and statistics!. J. Wound Care.

[3] van Laren L. (2012). Using HIV&AIDS statistics in pre-service Mathematics Education to integrate HIV&AIDS education. SAHARA J..

[4] Wilantari R.N., Latifah S., Wibowo W., Al Azies H. (2022). Additive mixed modeling of impact of investment, labor, education and information technology on regional income disparity: An empirical analysis using the statistics Indonesia dataset. Data Brief.

[5] Watt H.C. (2021). Reflection on modern methods: Statistics education beyond ‘significance’: Novel plain English interpretations to deepen understanding of statistics and to steer away from misinterpretations. Int. J. Epidemiol..

[6] Tay D. (2023). Turning metaphor on its head: A “target-to-source transformation” approach in statistics education. Front. Psychol..

[7] Senior C. (2016). Innovation in education. Commentary: Teaching statistics using dance and movement and a case for neuroscience in mathematics education. Front. Psychol..

[8] Schwartz T.A. (2014). Flipping the statistics classroom in nursing education. J. Nurs. Educ..

[9] Huang A., Henderson G., Profeta A., Pfeiffer M., Feinstein L.H., Delahunta M., LaHood C., Michael J.J., Mizia A.C., Levitsky D.A. (2023). Lack of compensation of energy intake explains the success of alternate day feeding to produce weight loss. Physiol. Behav..

[10] Huang S.Y., Johnathan R., Shah N., Srivastava P., Huang A.A., Gress F. (2023). Technical Report: Protocol for Characterizing Phenotype Variants Using Phenome-Wide Association Study (PheWAS) Utilizing the Nationwide Inpatient Sample 2020 in Individuals with Pancreatic Cysts and Lung Cancer. Cureus.

[11] Huang A.A., Huang S.Y. (2023). Dendrogram of transparent feature importance machine learning statistics to classify associations for heart failure: A reanalysis of a retrospective cohort study of the Medical Information Mart for Intensive Care III (MIMIC-III) database. PLoS ONE.

[12] Huang A.A., Huang S.Y. (2023). Computation of the distribution of model accuracy statistics in machine learning: Comparison between analytically derived distributions and simulation-based methods. Health Sci. Rep..

[13] Wright C., Meng Q., Breshock M.R., Atta L., Taub M.A., Jager L.R., Muschelli J., Hicks S.C. (2024). Open Case Studies: Statistics and Data Science Education through Real-World Applications. J. Stat. Data Sci. Educ..

[14] Smeeton N.C. (2008). Comments on ‘Statistical education for medical students—Concepts are what remain when the details are forgotten’ by Amir Herman, Netta Notzer, Zipi Libman, Rony Braunstein and David Steinberg. Statistics in Medicine 2007; 26:4344–4351. Stat. Med..

[15] Hancock M., Peulen T.-O., Webb B., Poon B., Fraser J.S., Adams P., Sali A. (2022). Integration of software tools for integrative modeling of biomolecular systems. J. Struct. Biol..

[16] Chu X., Wang Y., Tian P., Li W., Mercadante D. (2021). Editorial: Advanced Sampling and Modeling in Molecular Simulations for Slow and Large-Scale Biomolecular Dynamics. Front. Mol. Biosci..

[17] Andreani J., Jimenez-Garcia B., Ohue M. (2023). Editorial: Web tools for modeling and analysis of biomolecular interactions Volume II. Front. Mol. Biosci..

[18] Hempel T., Del Razo M.J., Lee C.T., Taylor B.C., Amaro R.E., Noe F. (2021). Independent Markov decomposition: Toward modeling kinetics of biomolecular complexes. Proc. Natl. Acad. Sci. USA.

[19] Andreani J., Ohue M., Jimenez-Garcia B. (2022). Editorial: Web Tools for Modeling and Analysis of Biomolecular Interactions. Front. Mol. Biosci..

[20] Gawthrop P.J. (2021). Energy-Based Modeling of the Feedback Control of Biomolecular Systems with Cyclic Flow Modulation. IEEE Trans. Nanobiosci..

[21] Ejalonibu M.A., Elrashedy A.A., Lawal M.M., Kumalo H.M., Mhlongo N.N. (2022). Probing the dual inhibitory mechanisms of novel thiophenecarboxamide derivatives against Mycobacterium tuberculosis PyrG and PanK: An insight from biomolecular modeling study. J. Biomol. Struct. Dyn..

[22] Schlick T., Portillo-Ledesma S., Myers C.G., Beljak L., Chen J., Dakhel S., Darling D., Ghosh S., Hall J., Jan M. (2021). Biomolecular Modeling and Simulation: A Prospering Multidisciplinary Field. Annu. Rev. Biophys..

[23] Pallesen P.B., Tverborgvik T., Rasmussen H.B., Lynge E. (2010). Data on education: From population statistics to epidemiological research. Scand. J. Public. Health.

[24] Masel J., Humphrey P.T., Blackburn B., Levine J.A. (2015). Evidence-Based Medicine as a Tool for Undergraduate Probability and Statistics Education. CBE Life Sci. Educ..

[25] Ramos R.L., Fokas G.J., Bhambri A., Smith P.T., Hallas B.H., Brumberg J.C. (2011). Undergraduate Neuroscience Education in the U.S.: An Analysis using Data from the National Center for Education Statistics. J. Undergrad. Neurosci. Educ..

[26] Franklin C. (2021). As Covid makes clear, statistics education is a must. Significance.

[27] Calin-Jageman R.J. (2017). After p Values: The New Statistics for Undergraduate Neuroscience Education. J. Undergrad. Neurosci. Educ..

[28] Kasson P.M. (2022). Modeling biomolecular kinetics with large-scale simulation. Curr. Opin. Struct. Biol..

[29] da Silva A.S., Barbosa M.T.S., de Souza Velasque L., da Silveira Barroso Alves D., Magalhaes M.N. (2021). The COVID-19 epidemic in Brazil: How statistics education may contribute to unravel the reality behind the charts. Educ. Stud. Math..

[30] Bellack J.P. (2014). Special focus on statistics education. J. Nurs. Educ..

[31] Agrawal S., Austin S. (2023). An idea to explore: Augmented reality and LEGO(R) brick modeling in the biochemistry and cell biology classroom-two tactile ways to teach biomolecular structure-Function. Biochem. Mol. Biol. Educ..

[32] Barclay A., Kragelund B.B., Arleth L., Pedersen M.C. (2023). Modeling of flexible membrane-bound biomolecular complexes for solution small-angle scattering. J. Colloid. Interface Sci..

[33] Turner S., Pham T., Robledo K., Turner S., Brown C., Sundaresan P. (2022). Rapid Adaptation of Cancer Education in Response to the COVID-19 Pandemic: Evaluation of a Live Virtual Statistics and Research Skills Workshop for Oncology Trainees. J. Cancer Educ..

[34] Rota M., Peveri G., Fanelli M., Torelli L., Rocchi M.B., Specchia C. (2021). Satisfaction with online teaching of medical statistics during the COVID-19 pandemic: A survey by the Education Committee of the Italian Society of Medical Statistics and Clinical Epidemiology. Teach. Stat..

[35] Jung Y., Sadeghi A., Ha B.Y. (2024). Modeling the compaction of bacterial chromosomes by biomolecular crowding and the cross-linking protein H-NS. Sci. Rep..

